# Machine learning models including patient-reported outcome data in oncology: a systematic literature review and analysis of their reporting quality

**DOI:** 10.1186/s41687-024-00808-7

**Published:** 2024-11-05

**Authors:** Daniela Krepper, Matteo Cesari, Niclas J. Hubel, Philipp Zelger, Monika J. Sztankay

**Affiliations:** 1 Department of Psychiatry, Psychotherapy, Psychosomatics and Medical Psychology, University Hospital of Psychiatry II, Medical University of Innsbruck, Innsbruck, Austria; 2 Department of Neurology and Neurosurgery, Medical University of Innsbruck, Innsbruck, Austria; 3 University Hospital for Hearing, Speech & Voice Disorders, Medical University of Innsbruck, Innsbruck, Austria

**Keywords:** Patient-reported outcomes, Oncology, Machine learning, Artificial intelligence, Deep learning

## Abstract

**Purpose:**

To critically examine the current state of machine learning (ML) models including patient-reported outcome measure (PROM) scores in cancer research, by investigating the reporting quality of currently available studies and proposing areas of improvement for future use of ML in the field.

**Methods:**

PubMed and Web of Science were systematically searched for publications of studies on patients with cancer applying ML models with PROM scores as either predictors or outcomes. The reporting quality of applied ML models was assessed utilizing an adapted version of the MI-CLAIM (Minimum Information about CLinical Artificial Intelligence Modelling) checklist. The key variables of the checklist are study design, data preparation, model development, optimization, performance, and examination. Reproducibility and transparency complement the reporting quality criteria.

**Results:**

The literature search yielded 1634 hits, of which 52 (3.2%) were eligible. Thirty-six (69.2%) publications included PROM scores as a predictor and 32 (61.5%) as an outcome. Results of the reporting quality appraisal indicate a potential for improvement, especially in the areas of model examination. According to the standards of the MI-CLAIM checklist, the reporting quality of ML models in included studies proved to be low. Only nine (17.3%) publications present a discussion about the clinical applicability of the developed model and reproducibility and only three (5.8%) provide a code to reproduce the model and the results.

**Conclusion:**

The herein performed critical examination of the status quo of the application of ML models including PROM scores in published oncological studies allowed the identification of areas of improvement for reporting and future use of ML in the field.

**Supplementary Information:**

The online version contains supplementary material available at 10.1186/s41687-024-00808-7.

## Introduction

The amount of data produced by clinical trials, research projects, and patient care in oncology has grown exponentially in recent years [1]. A valuable part of this data contains patient-reported outcomes (PROs), such as quality of life, that directly provide the patients’ views on the impact of disease and treatment on their health status without prior interpretation by clinicians [2]. In oncology, PROs are recognized as critical endpoints for assessing the effectiveness of cancer treatments and interventions, particularly considering the calls for patient involvement and patient-centeredness [3]. Potential clinical benefits of PROs range from enhanced symptom management and patient-clinician communication to improved overall survival [4, 5]. The growing availability of PRO data harbors both benefits and challenges. Large datasets can offer insightful information on the underlying mechanisms of cancer and prospective therapeutic targets [6]. However, complex intelligent system tools that go beyond conventional statistical methods are needed to analyze this data. In this context, artificial intelligence (AI) algorithms are increasingly being used in medical research and applications, as they allow for the analysis of big data with reliability in a variety of medical fields, from radiology to genomics [7–10]. Incorporating patient-reported outcome measures (PROMs) into AI studies humanizes AI in healthcare by integrating patients’ perspectives. This approach offers a comprehensive view of health, aiding collaborative decision-making beyond solely relying on traditional clinical endpoints like survival [6]. Machine Learning (ML) is a subfield of AI and consists of computer algorithms that are trained to identify patterns in predictors (also called “features” in ML terminologies) and make predictions based on them. ML algorithms use various types of learning strategies, of which the most common are supervised and unsupervised learning. The ML type covered in this review incorporates supervised ML algorithms trained to predict predefined outcomes, by learning the patterns in the data and mapping predictors to desired outputs. After training, supervised ML models are tested on unseen data, and the performances of the prediction are evaluated. Unsupervised ML algorithms, instead, are trained without knowing the desired output. Unsupervised learning is typically used when researchers want to investigate new patterns in complex data. Deep learning (DL) is a subfield of ML and includes deep artificial neural networks, i.e., artificial neural networks composed of several layers of artificial neurons. Compared to classical ML algorithms, DL algorithms usually require significantly more training data and computation power [11].

The increasing interest in ML in medicine, with a particular focus on supervised ML, requires methodological rigor in its application and reporting. The algorithms need to be trained and tested using appropriate datasets and methodology to ensure that the results and models are valid, reliable, and generalizable to the wider patient population [12–15].

To offer an overview of published oncological studies reporting ML algorithms including PROM scores, the objectives of this systematic review were threefold:

1. To assess whether there has been an increase in research publications about ML with PROM scores as a predictor or an outcome over the last years.
2. To assess which ML algorithms and PROMs are most commonly used in the field of interest.
3. To evaluate the quality of the reporting of applied ML models - according to a modified version of the “minimum information about clinical artificial intelligence modeling” (MI-CLAIM) [16] checklist.

## Methods

### Registration and reporting guideline

The review was registered with Prospero (ID: CRD42023405660) and reported using the Preferred Reporting Items for Systematic Reviews and Meta-Analyses (PRISMA) guideline [17].

### Information sources

We searched Pubmed and Web of Science for studies applying ML methods including PROM scores as a predictor and/or as an outcome within the oncology field and published until 13 December 2022 (the date of search). No filters were applied. The full search strategies and documentation are provided in Supplementary Tables S1 and S2. The database search was complemented with a backward search of review publications identified in the screening process.

### Eligibility criteria

References were excluded if they were reviews, guidance documents, or lacked a patient population diagnosed with cancer (excluding mixed populations with cancer and other diseases). It led to exclusion if publications did not involve supervised machine learning or deep learning models or did not include PROM scores as either a predictor or an outcome. Finally, references were only eligible if they were in English, and the full text was available to the authors.

### Study selection and data extraction

The literature review software DistillerSR [18] was used to provide full transparency of the 2-level study selection process and the subsequent data extraction. Two reviewers from a combination of three (DK, MC, NJH) worked independently to decide on the eligibility of each record on the abstract and full-text level and solved emerging conflicts. If no agreement was reached, a third reviewer (MS) was consulted. General study characteristics were extracted by one reviewer (DK). Data to answer the a priori-defined review questions on the quality of reporting of applied ML models were extracted by two reviewers (MC & PZ), who are technical experts in the ML field. Disagreements were discussed by these two reviewers.

### Data items (list of outcomes/variables extracted)

Descriptive information, including cancer type, country, and sample size was extracted. The PROMs used were recorded, and it was evaluated whether PROM scores were included as predictors or outcomes in the respective ML model. Information on the type of ML model, its aim, the ratio of cross-validation training set to test set, and the best result were gathered.

### Assessment of the quality of reporting

The quality of reporting appraisal followed a prespecified procedure, using an adapted version of the Minimum Information about CLincal Artificial Intelligence Modeling (MI-CLAIM) checklist [16]. This checklist was developed with the purpose of assessing both the clinical impact, including fairness and biases, of an AI model and the replication of its technical design [16]. The applied procedure was adapted from a previous study [19]. Unlike to the work by Smets et al. [19], we removed two questions on comparison with state-of-the-art baseline methods, as it was not possible to identify baseline methods in this field of research. Standard operating procedures were established a priori to guarantee a common understanding of variables. In Table 1 the adapted MI-CLAIM checklist is reported, including comments proposed in the publication by the authors [19], which guided the interpretation of the items. The checklist is composed of 10 items, which assess the quality of the reporting for (i) study design, (ii) data preparation and partitioning, (iii) model development, optimization, and final model selection, (iv) model performance, (v) model examination and (vi) reproducibility and transparency.

**Table 1:** MI-CLAIM checklist for quality of reporting appraisal (modified from [16, 19])

Category/Item description	Comments
**Study design**	
Research task: Research task is clearly stated, including the intended clinical problem, specified input predictors and an identified outcome	Research task defined with intended problem to solve, with known input and output numbers
Data characteristics: The characteristics of the dataset (training and test sets if applicable) are detailed and have been shown to be representative to the research settings.	Characteristics of the input and output data is precise and is appropriate to estimate its practical application
**Data preparation and partitioning**	
Data transformation: Transformations of the data before it is applied to the proposed model are described (e.g., missing data imputation, normalization, feature selection, etc.)	All transformations are clearly described allowing data formatting for replication
Validation methodology: The validation methodology is clearly explained.	Use of separate splits, cross-validation, etc.
Train/test independence: The independence between training and test sets has been proven in the paper.	No patients overlapping is demonstrated
**Model development, optimization and final model selection**	
Model configuration and parameters: Details on the models that were evaluated, and the adequate method developed to select the best model are provided.	Full and clear description of each developed model with their hyperparameters (reasons for this selection are not required)
**Model performance**	
Performance metrics: An appropriate primary metric selected to evaluate algorithm performance (e.g.: AUC, F-score, etc.) has been clearly stated and reported.	Evaluation metric reported with numerical values (reasons for this selection are not required)
**Model examination**	
Examination technique: An adequate examination technique has been reported (e.g., feature importance, sensitivity analysis, saliency maps, etc.)	Examination technique allowing clearer understanding of the model decision-making process or implication in practice (also include permutation feature importance)
Reliability and robustness discussion: A discussion of the reliability and robustness of the model is included and limitations for its clinical use have been clearly described.	Objective discussion on strength and weaknesses of the study
**Reproducibility and transparency**	
Reproducibility and transparency: A link to the model development code, the final developed model or the data have been reported allowing replication.	Either development code, final developed model or data has been shared

### Synthesis methods

Frequencies and respective percentages of categorical variables (e.g., cancer type, country, PROM used) were calculated using SPSS v. 29.0.0.0. The calculation of the “ML Quality of Reporting Score” (MLQRS) followed this scheme: “completed” = 1 point and “not completed” = 0. The maximum achievable score was 10.

## Results

**Fig. 1:**
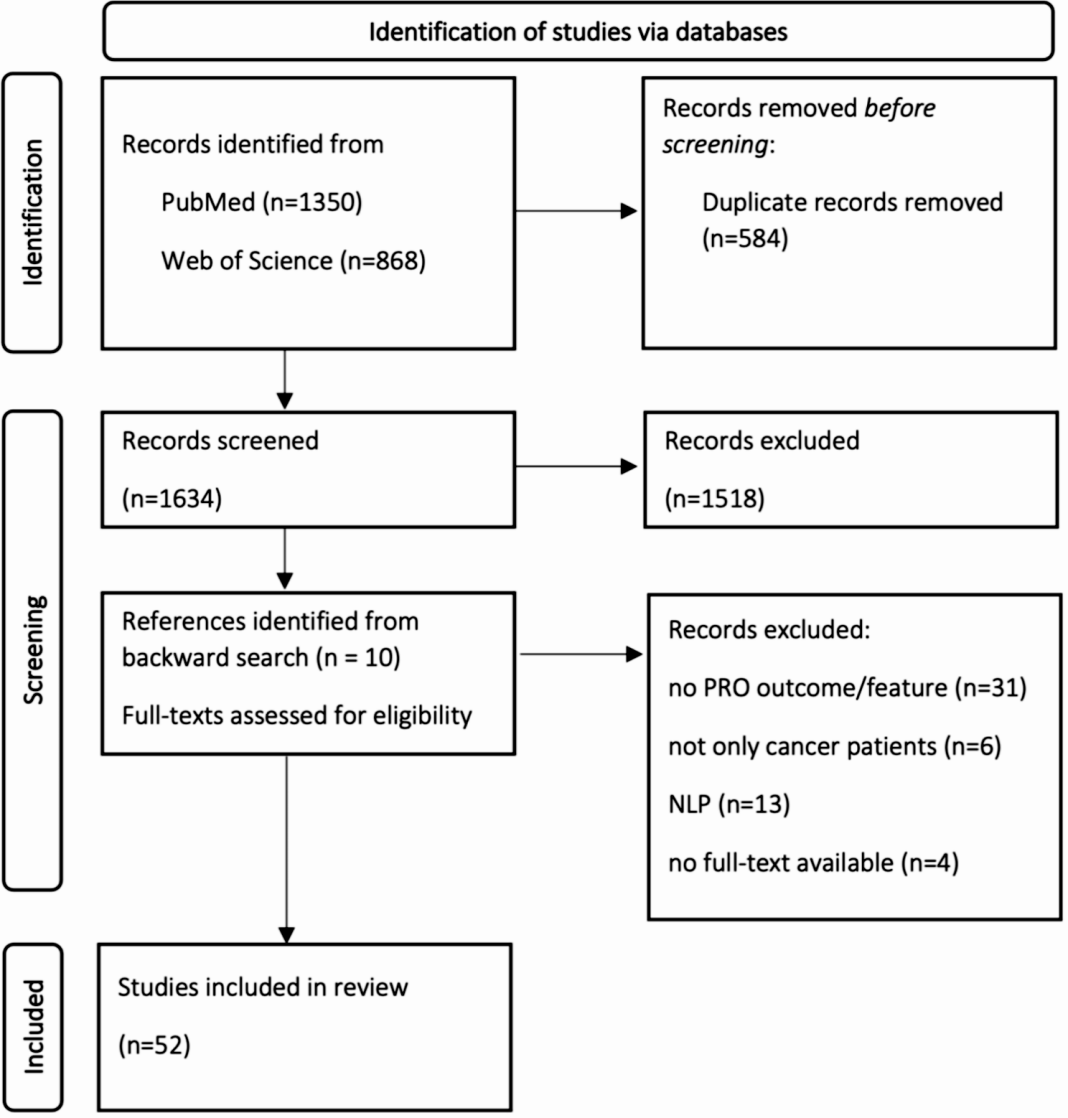
Flow chart (adapted from Page et al. [17])

The search resulted in 1634 unique references that were screened for eligibility. The two-stage selection procedure resulted in 52 (3.2%) studies considered eligible for the review. For full traceability, Fig. 1 shows the total study selection process.

### Study characteristics

**Fig. 2:**
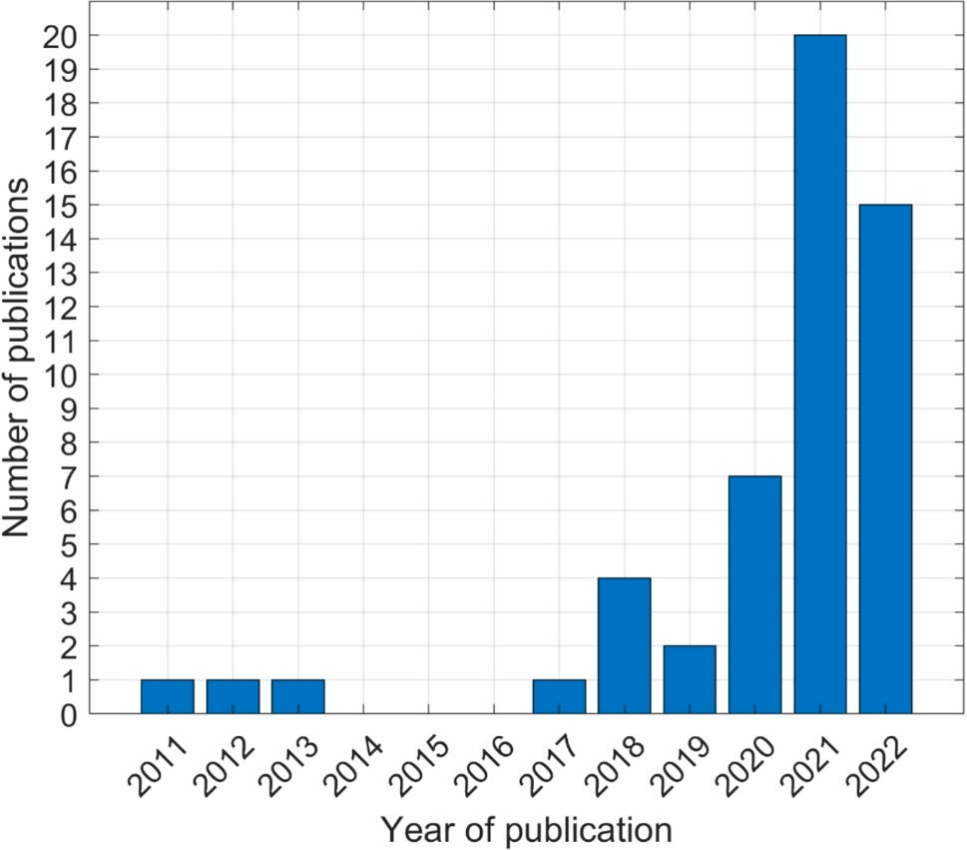
Year of publication of included studies

Twenty-five of the reviewed studies (48.1%) included patients with breast cancer, being the most frequent cancer type examined followed by lung cancer (*n* = 13, 25.0%), and gastrointestinal cancers (*n* = 11, 21.2%). Country-wise, the majority of studies were conducted in the USA (*n* = 21, 40.4%). In total, the evaluated studies included 102 977 patients (IQR = 818, min = 25, max = 46104). Thirty-six (69.2%) studies included PROM scores as a predictor and 32 (61.5%) as an outcome. The most frequently used PRO questionnaires were measures provided by the European Organisation for Research and Treatment of Cancer (EORTC) (e.g., [20]) with ten (19.2%) studies using them, followed by the Short Form 12 or 36 (SF 12/36) [21, 22] (*n* = 6, 11.5%), and the Edmonton Symptom Assessment System (ESAS) [23] (*n* = 5, 9.6%). A comprehensive overview of the study characteristics of the included studies is presented in Table 2. Figure 2 illustrates the number of included publications per year.

**Table 2:** Study characteristics (*N* = 52)

	*N*	%
Type of cancer*		
Breast cancer	25	48.1%
Lung cancer	13	25.0
Gastrointestinal cancers	11	21.2
Prostate cancer	10	19.2
Gynecological cancers**	8	15.4
Head and neck cancers	8	15.4
Colorectal cancer	7	13.5
Skin cancer***	4	7.7
Esophaegeal cancer	3	5.8
Hematological cancers	3	5.8
Kidney (renal) cancer	3	5.8
Bladder cancer	2	3.8
Neurological cancers	2	3.8
Pancreatic cancer	2	3.8
Thyroid cancer	2	3.8
Other	6	11.5
Unclear	5	9.6
Country*		
US	21	40.4
Canada	5	9.6
China, Finland, Japan, Taiwan	4 (each)	7.7
Italy	3	5.8
Israel, Korea, Portugal	2 (each)	3.8
Brazil, France, Germany, Greece, Indonesia, Malaysia, Romania, Serbia, Türkiye	1 (each)	1.9
**Sample size**		
1–100	10	19.2
101–300	10	19.2
301–999	19	36.5
>1000	13	25.0
PROM used*		
EORTC questionnaires (C30 and/or modules)	10	19.2
SF-12/36	6	11.5
ESAS	5	9.6
FACIT questionnaires^a^	4	7.7
HADS	4	7.7
Pain (VAS/NRS)	4	7.7
PROMIS questionnaires^b^	4	7.7
STAI	4	7.7
CES-D	3	5.8
EPIC-26	3	5.8
EQ-5D	3	5.8
GAD	3	5.8
MDASI	3	5.8
BDI	2	3.8
CD-RISC	2	3.8
PHQ	2	3.8
PRO-CTCAE	2	3.8
Other	25	48.1
Unclear	4	7.7

*Abbreviations SF-12/36* Short Form 12/36, *ESAS* Edmonton Symptom Assessment System, *FACIT* Functional Assessment of Chronic Illness Therapy, *HADS* Hospital Anxiety and Depression Scale, *PROMIS* Patient-Reported Outcomes Measurement Information System, *STAI* State-Trait-Anxiety Inventory, *CES-D* Center for Epidemiologic Studies Depression Scale, *EPIC-26* Expanded Prostate Cancer Index Composite 26, *EQ-5D* EuroQol-5D, *GAD* Generalised Anxiety Disorder Assessment, *MDASI* MD Anderson Symptom Inventory, *BDI* Beck’s Depression Inventory, *CD-RISC* Connor-Davidson Resilience Scale, *PHQ* Patient Health Questionnaire, *PRO-CTCAE* Patient-Reported Outcomes version of the Common Terminology Criteria for Adverse Events

^a^FACT-G, FACT-Hep, FACT-F, FACT-Cog, FACT-OC

^b^PROMIS-29, PROMIS Global, PROMIS Interest in Sexual Activity and Global Satisfaction with Sex Life, PROMIS global physical and mental health scales

*Multiple entry possible

**Cervical, ovarian/fallopian tubes/peritoneum, uterine, vaginal, vulvar

***Melanoma and non-melanoma

### Type and quality of reporting of applied ML models

Artificial neural networks were the ML algorithm mainly used in the included studies (*n* = 14, 26.9%), followed by random forest classifiers (*n* = 11, 21.2%). In the 36 studies where PROM scores were used as predictors to an ML model, the aim was mainly to predict other PROM scores (*n* = 16, 44.4%) and survival/mortality (*n* = 11, 30.6%).

**Fig. 3:**
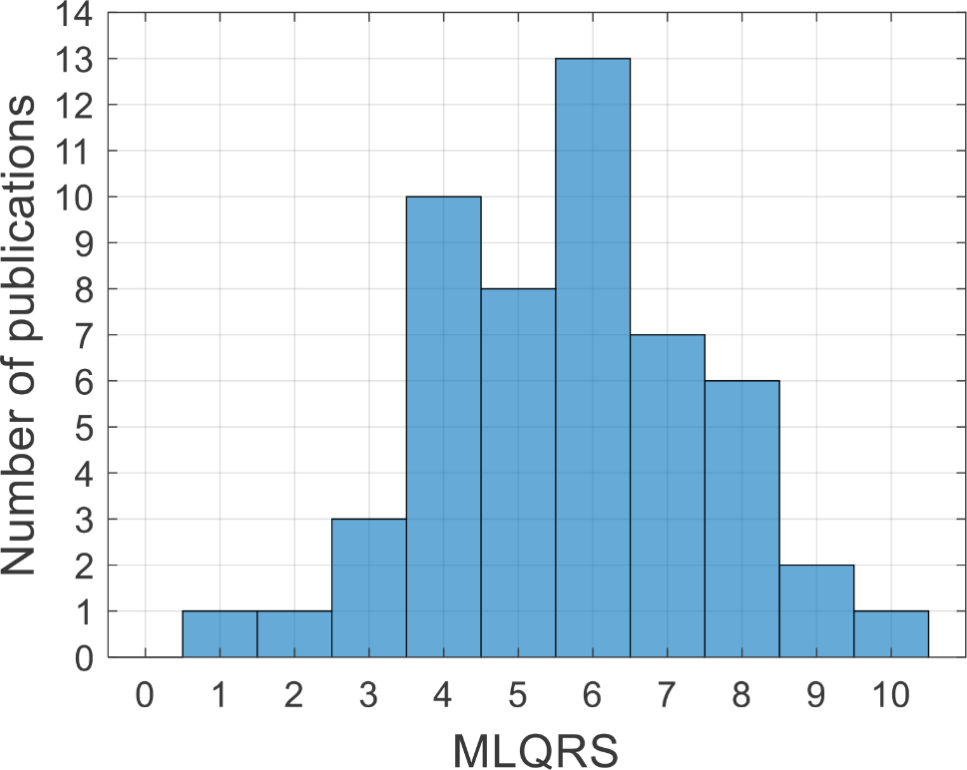
Distribution of machine learning quality of reporting score (MLQRS) across the publications

Only 9 (17.3%) of the included studies had an MLQRS higher or equal than 8, while the majority of studies had an MLQRS between 5 and 7 (*n* = 28, 53.8%). Concerning single items of quality of reporting scores (see Supplementary Table S3 for the results on an individual study-level), only a minority of studies clearly described data transformation (*n* = 13, 25%), detailed model configuration, and hyperparameters (*n* = 16, 30.8%), objectively discussed the reliability and robustness of the model (*n* = 9, 17.3%) and provided code, pseudo-code, or data (*n* = 3, 5.8%). See Table 3 for a full overview of ML-related results, Fig. 3 for a histogram portraying the distribution of the MLQRS across studies, and Supplementary Table S4 for individual study-level results for both, PRO and ML variables combined.

**Table 3:** Machine-learning related results (*N* = 52)

	*N*	%
**PRO included in the model as a**(*n*)*		
Predictor	36	69.2
Outcome	32	61.5
ML algorithm**		
Artificial neural network (no deep neural networks)	14	26.9
Random forest	11	21.2
Boosting methods	9	17.3
Regression algorithms	6	11.5
Ensemble of several ML algorithms	4	7.7
Decision trees	3	5.8
Deep learning (CNN and LSTM)	3	5.8
Extra tree classifier	1	1.9
Support vector machine	1	1.9
**Aim of ML model when PROs used as predictor (** ***N*** ** = 36)**		
Predict other PROs	16	44.4
Predict survival	11	30.6
Predict adverse/acute events	3	8.3
Other	6	16.7
**Overall MLQRS**		
>=8	9	17.3
5–7	28	53.8
<=4	15	28.8
**Single quality of reporting items**		
Research task	48	92.3
Data characteristics	35	67.3
Data transformations	13	25.0
Validation methodology	40	76.9
Train/test independence	42	80.8
Model configuration and parameters	16	30.8
Performance metric	49	94.2
Examination technique	39	75.0
Reliability and robustness discussion	9	17.3
Reproducibility and transparency	3	5.8

*PRO* patient reported outcome, *ML* machine learning, *CNN* convolutional neural network, *LSTM* long-short term memory

*Multiple entry possible

**In some publications, several ML models have been tested. In this case, only the ML model leading to the best classification performances has been reported in the table

## Discussion

The Artificial Intelligence Index Report 2021 states that the number of peer-reviewed AI publications grew by nearly 12 times between 2000 and 2019 [24], revealing the hype the field has experienced in the last two decades. The development is mirrored in the niche of PRO research in oncology, covered in this review. The number of publications reporting ML models including PROM scores increased 20-fold between 2011 and 2021. The trend continued, albeit at a slower pace, through 2022. The predominance of the measurement system of the EORTC aligns with a recent review [25] showing that more than half of cancer clinical trials in six main cancer populations published between 2014 and 2019 used EORTC PROMs. Looking at the architecture of included ML studies, most of these included in the review employed artificial neural networks (ANN), not designed as deep neural networks, as the final model. It is also interesting to observe that out of 16 studies where ANN and multiple other algorithms were tried, ANN was selected as the best classifier in 9 (56.3%) and was part of the final ensemble model in another four works (25%). Although ANNs are generally more computational expensive than other simpler algorithms, they can detect complex and non-linear relationships between inputs and outputs, making them potentially superior to other methods. Similarly, random forest (RF), the second most employed algorithm in the included studies, is an ensemble of decision trees that typically outperforms single classifiers [26].

In contrast to current ML research in cancer prediction and diagnosis, where more models are based on deep learning models [27], only three of the included studies proposed deep neural network architectures. The cause of this can be manifold. First, PROM scores have the typical data structure of features (i.e., structured variables to be given in input to a model), which perhaps influenced researchers opting more for classical ML methods than for DL algorithms, which are typically more suited for input data like images or signals. Second, the sample size of the included studies was generally low and DL algorithms usually require a big enough sample size to be properly trained and validated. Third, because of their complexity, it is more difficult to understand how DL models make predictions and decisions compared to classical ML ones [28]. In the medical field, interpretability is a crucial factor in including ML models in clinical work [29]. Although many research groups have developed methods to try to explain the decision process of DL models, such methods are not univocally accepted by the medical community [30]. On the other side, feature importance and sensitivity analyses provide a better understanding of the decision-making process of ML models [28]. The majority of the studies included in the review used these approaches to explain the decision-making process of the proposed models. With the increasing size and complexity of datasets, it is likely that shortly the field will move to investigate deep learning architectures to evaluate PROMS in oncology. In particular, natural language processing methods to analyze both structured as well as unstructured PROM scores might be a promising way to explore [31]. In the field of natural language processing, there is also a great advancement in the field of interpretability and explainability, which might facilitate not only the investigation of these models but also their future integration [32].

With a mean quality of reporting score of 5.7 across the included studies, this work shows that, overall, the quality of reporting of ML models is relatively low, similar to what was shown in related work in the field of oncology without any PRO focus [33]. The results of another recent review indicate a high risk of bias in prognostic models using ML in oncology [34]. A common issue affecting most of the included publications concerns reproducibility, as details on data transformations, model parameters, and open-source code were available only for the minority of the studies. The lack of reproducibility of ML models including PROM scores in medical research is a known problem and strategies to overcome it should be implemented as soon as possible [35]. Furthermore, only very few studies (7.7%) validated their models on external datasets. External validation means that a trained ML model is applied to a different population or setting [15]. To integrate ML models in clinical practice, such external validation is essential, as only through this step it is possible to ensure that a model is generalizable to a new population and that it does not suffer from either overfitting, unfairness, or bias [15]. This is particularly important for the models included in this review, which were generally trained in small datasets. External validation is particularly critical in oncology, where patient populations, treatment regimens, and healthcare settings vary considerably [36].

Without transparent and high-quality reporting, it is hard to understand whether the models might be affected by unfairness and biases. As an example, the lack of a clear description that the dataset is diverse and representative of different conditions (e.g. patients with different ages, genders, and ethnicities) might reflect the development of a biased model. When applied to an underrepresented population with different characteristics, this model might perform differently thanexpected, affecting patient outcomes and raising ethical questions [37].

As reproducibility and external validations are prerequisites for the implementation of ML algorithms in clinical practice [38] and due to the relatively low quality of reporting of the ML models included in this work, our study suggests that the integration of the proposed models in clinical practice is still far to be achieved. The availability of tools such as the MI-CLAIM [16] should guide researchers to improve the quality of reporting of ML algorithms.

### Limitations of the review processes applied

The MI-CLAIM checklist has been developed with the aim of evaluating the reporting quality of ML algorithms and should not be therefore seen as a tool to evaluate the quality of the ML modelling itself. As the ML algorithms included in this systematic review address different research questions and use different datasets, a direct comparison of their quality in terms of classification performances was not possible.

As an additional limitation, we also noticed that the interpretation of the MI-CLAIM checklist is not always objective and univocal. We found it particularly difficult to objectively evaluate the presence of a clear discussion of the reliability and robustness of the model (point 9 in the checklist). Furthermore, we excluded studies proposing unsupervised learning models, as some items of the MI-CLAIM checklist (Validation methodology, Train/test independence, and Performance metrics) were not applicable, as already reported in [19].

One limitation of this review is that it relied on only two major databases for the literature search. While these databases were selected for their extensive coverage of relevant studies, including additional databases could have provided a broader scope and potentially captured additional relevant literature.

### Implications of results for practice, policy, and future research

With regulatory bodies starting to systematically emphasize the use of ML in medical research and drug development [39, 40] and the AI act of the European Union coming into effect [41], the quality of reporting of ML models including PROM scores needs to be prioritized. The herein performed critical examination of the status quo of ML models including PROM scores in oncology allowed us to identify areas of improvement for future reporting of ML in the field, in particular concerning the reproducibility of the models. Improving the reproducibility of ML models would allow independent research groups to externally validate the ML models on new datasets, to evaluate their generalizability and robustness to novel populations and settings [42]. Only with external validations, ML models can be considered reliable and applicable in clinical contexts, as seen in other fields [43]. The relatively small size of databases employed in the studies included in this review calls also for the need for more collaborative efforts in the development and validation of ML algorithms including PROM scores in oncology. Sharing data open access, following the FAIR principles [44], as well as using a standardized minimum set of PROMs in clinical oncology studies [45], would also allow researchers to quickly have access to plenty of large datasets for robust ML model development. Only with large and multicentric data reliable ML models can be developed and, possibly, applied in clinical practice soon.

Based on the results of this systematic review, researchers should, at a bare minimum, use the MI-CLAIM [16] checklist in future documentation of ML models. To further improve the quality of current ML research, journals might also consider asking their reviewers to check the quality of reporting of ML models by applying available study-specific reporting guidelines that are recognized by the scientific community (e.g., CONSORT-AI for randomized controlled trials, SPIRIT-AI for protocols,…) [46]. Furthermore, due to the increasing number of applications of AI in medicine, medical journals should seek to involve engineers, computer scientists, or professionals who have a methodological understanding of ML in their editorial boards. Including respective professionals in the review process of these manuscripts would allow checking for the basic requirements of proper development and reporting of ML models. As an example, among the studies included in this systematic review, ten did not clearly prove the independence between training and testing data sets, which is a fundamental aspect of ensuring the quality of applied ML.

## Conclusion

Facing the increasing amount of available data, including PROM scores, with the potential to benefit both clinical research and practice, we lean on novel technology. ML algorithms are a promising tool to learn complex patterns and provide estimates based on PRO data. To meet the full potential of this technology in a thriving field and to ensure that ML models provide researchers, clinicians, and patients with valid and reliable results, transparent reporting is critical given their possible future integration into clinical practice.

## Electronic supplementary material

Below is the link to the electronic supplementary material.

Supplementary Material 1

## Data Availability

Not applicable.
