# Supplementary material for: Machine learning models including patient-reported outcome data in oncology: a systematic literature review and analysis of their reporting quality
Source: J Patient Rep Outcomes. 2024 Nov 5;8:126. doi: 10.1186/s41687-024-00808-7 (PMC11538124; doi:10.1186/s41687-024-00808-7)
Supplement: Supplementary file 1 — Supplementary Material 1 [file 41687_2024_808_MOESM1_ESM.docx]

*APPENDIX*

Search strategies

Date of search Pubmed: 13.12.2022

Filter: none

Supplementary Table S 1 Search Strategy & Documentation Pubmed

| # |  | hits |
| --- | --- | --- |
| #1 | ("quality of life"[MeSH Terms] OR "quality of life"[Title/Abstract]) OR ("health related quality of life"[Title/Abstract]) OR ("life quality"[Title/Abstract]) OR ("health outcomes"[Title/Abstract]) OR ("health status"[Title/Abstract]) OR ("patient reported symptom"[Title/Abstract]) OR ("patient reported outcomes"[Title/Abstract]) OR ("patient reported outcome"[Title/Abstract]) OR ("PRO"[Title/Abstract]) OR ("PROs"[Title/Abstract]) OR ("HRQL"[Title/Abstract]) OR ("QOL"[Title/Abstract]) OR ("HRQOL"[Title/Abstract]) OR ("self report"[Title/Abstract]) OR ("self assessment"[Title/Abstract]) OR ("self disclosure"[Title/Abstract]) | 868,827 |
| #2 | "Artificial intelligence"[MeSH Terms] OR "machine-learning"[MeSH Terms] OR "neural networks, computer"[MeSH Terms] OR "machine learning"[Title/Abstract] OR "machine-learning"[Title/Abstract] OR "artificial intelligence"[Title/Abstract] OR "AI"[Title/Abstract] OR "deep-learning"[Title/Abstract] OR "deep-learning"[Title/Abstract] OR "deeplearning"[Title/Abstract] OR "neural network*"[Title/Abstract] | 293,606 |
| #3 | "cancer s"[All Fields] OR "cancerated"[All Fields] OR "canceration"[All Fields] OR "cancerization"[All Fields] OR "cancerized"[All Fields] OR "cancerous"[All Fields] OR "neoplasms"[MeSH Terms] OR "neoplasms"[All Fields] OR "cancer"[All Fields] OR "cancers"[All Fields] OR "cysts"[MeSH Terms] OR "cysts"[All Fields] OR "cyst"[All Fields] OR "neurofibroma"[MeSH Terms] OR "neurofibroma"[All Fields] OR "neurofibromas"[All Fields] OR "tumor s"[All Fields] OR "tumoral"[All Fields] OR "tumorous"[All Fields] OR "tumour"[All Fields] OR "neoplasms"[MeSH Terms] OR "neoplasms"[All Fields] OR "tumor"[All Fields] OR "tumour s"[All Fields] OR "tumoural"[All Fields] OR "tumourous"[All Fields] OR "tumours"[All Fields] OR "tumors"[All Fields] OR "cysts"[MeSH Terms] OR "cysts"[All Fields] OR "cyst"[All Fields] OR "neurofibroma"[MeSH Terms] OR "neurofibroma"[All Fields] OR "neurofibromas"[All Fields] OR "tumor s"[All Fields] OR "tumoral"[All Fields] OR "tumorous"[All Fields] OR "tumour"[All Fields] OR "neoplasms"[MeSH Terms] OR "neoplasms"[All Fields] OR "tumor"[All Fields] OR "tumour s"[All Fields] OR "tumoural"[All Fields] OR "tumourous"[All Fields] OR "tumours"[All Fields] OR "tumors"[All Fields] | 5,321,638 |
| #4 | #1 AND #2 AND #3 | **1,350** |

Date of search Web of Science: 13.12.2022

Filter: none

Supplementary Table S 2 Search Strategy & Documentation Web of Science

| # |  | hits |
| --- | --- | --- |
| #1 | AB=("quality of life" OR "health related quality of life" OR "life quality" OR "health outcomes" OR "health status" OR "patient reported symptom" OR "patient reported outcomes" OR "patient reported outcome" OR PRO OR PROs OR HRQL OR QOL OR HRQOL OR "self report" OR "self assessment" OR "self disclosure") | 842,905 |
| #2 | AB=("machine learning" OR "machine-learning" OR "Artificial intelligence" OR "AI" OR "deep-learning" OR "deep-learning" OR "deeplearning" OR "neural network*") | 772,182 |
| #3 | ALL=("cancer*" OR "neoplasms" OR "neurofibroma*" OR "tumor*" OR "tumour*" OR "cyst*") | 5,291,861 |
| #4 | #1 AND #2 AND #3 | **868** |

Supplementary Table S 3 Study-level results: Quality of reporting score

| **Author (year)** | **I1** | **I2** | **I3** | **I4** | **I5** | **I6** | **I7** | **I8** | **I9** | **I10** | **MLQRS** |
| --- | --- | --- | --- | --- | --- | --- | --- | --- | --- | --- | --- |
| Walczak & Velanovich (2018) | 1 | 1 | 0 | 1 | 1 | 0 | 1 | 1 | 1 | 0 | 7 |
| Etminani-Ghasrodashti et al (2021) | 1 | 1 | 0 | 1 | 1 | 0 | 1 | 1 | 0 | 0 | 6 |
| Sharifi et al (2022) | 1 | 1 | 0 | 1 | 1 | 0 | 1 | 1 | 1 | 0 | 7 |
| Nuutinen et al (2021) | 1 | 1 | 0 | 1 | 1 | 0 | 1 | 1 | 0 | 0 | 6 |
| Etminani-Ghasrodashti et al (2021) | 1 | 1 | 0 | 1 | 1 | 0 | 1 | 1 | 0 | 0 | 6 |
| Hatzilygeroudis et al (2021) | 1 | 0 | 0 | 1 | 1 | 0 | 1 | 0 | 0 | 0 | 5 |
| Shi et al (2012) | 1 | 1 | 0 | 1 | 1 | 1 | 1 | 1 | 1 | 0 | 8 |
| Tsai et al (2013) | 1 | 0 | 0 | 0 | 0 | 1 | 1 | 1 | 0 | 0 | 4 |
| Takehira et al (2011) | 1 | 0 | 0 | 1 | 1 | 1 | 0 | 0 | 0 | 0 | 4 |
| Cvetković (2017) | 1 | 0 | 0 | 0 | 0 | 0 | 1 | 0 | 0 | 0 | 2 |
| Lötsch et al (2017) | 1 | 1 | 1 | 0 | 0 | 1 | 1 | 1 | 1 | 0 | 7 |
| Lee et al (2018) | 1 | 0 | 0 | 1 | 1 | 0 | 1 | 1 | 0 | 0 | 5 |
| Chiu et al (2018) | 1 | 1 | 0 | 1 | 1 | 0 | 1 | 1 | 0 | 0 | 6 |
| Sasani et al (2019) | 1 | 0 | 1 | 0 | 0 | 0 | 1 | 1 | 0 | 0 | 4 |
| Sim & Yun (2019) | 1 | 0 | 0 | 0 | 0 | 0 | 0 | 0 | 0 | 0 | 1 |
| Xu et al (2021) | 1 | 0 | 0 | 1 | 1 | 0 | 1 | 1 | 0 | 0 | 5 |
| Pan et al (2020) | 1 | 1 | 0 | 1 | 1 | 0 | 1 | 1 | 0 | 0 | 6 |
| Juwara et al (2020) | 1 | 1 | 0 | 0 | 0 | 0 | 1 | 1 | 0 | 0 | 4 |
| Sim et al (2020) | 1 | 0 | 0 | 0 | 0 | 0 | 1 | 1 | 1 | 0 | 4 |
| Arkin et al (2020) | 1 | 1 | 0 | 0 | 0 | 0 | 1 | 0 | 0 | 0 | 3 |
| Shafiei et al (2020) | 1 | 1 | 0 | 0 | 0 | 1 | 1 | 0 | 0 | 0 | 4 |
| Yang et al (2021) | 1 | 0 | 1 | 1 | 1 | 0 | 1 | 0 | 0 | 0 | 5 |
| Lou et al (2020) | 0 | 1 | 0 | 1 | 1 | 0 | 1 | 1 | 0 | 0 | 5 |
| Xuyi et al (2021) | 1 | 1 | 0 | 1 | 1 | 0 | 1 | 0 | 0 | 0 | 5 |
| Lee et al (2020) | 1 | 1 | 0 | 1 | 1 | 0 | 1 | 1 | 0 | 0 | 6 |
| Kourou et al (2021) | 1 | 1 | 1 | 1 | 1 | 1 | 1 | 1 | 0 | 0 | 8 |
| Rossi et al (2021) | 1 | 1 | 1 | 1 | 1 | 0 | 1 | 1 | 0 | 0 | 7 |
| Wang et al (2021) | 1 | 1 | 0 | 1 | 1 | 0 | 1 | 0 | 0 | 0 | 5 |
| Sidey-Gibbons et al (2021) | 1 | 1 | 1 | 1 | 1 | 1 | 1 | 1 | 0 | 0 | 8 |
| Haun et al (2021) | 1 | 1 | 1 | 1 | 1 | 1 | 1 | 1 | 0 | 0 | 8 |
| Wakabayashi et al (2021) | 1 | 1 | 0 | 1 | 1 | 1 | 1 | 0 | 0 | 0 | 6 |
| Iivanainen et al (2021) | 0 | 0 | 0 | 1 | 1 | 0 | 1 | 1 | 0 | 0 | 4 |
| Kober et al (2021) | 1 | 1 | 1 | 1 | 1 | 1 | 1 | 1 | 0 | 0 | 8 |
| Crumpei-Tanasă & Crumpei (2021) | 0 | 0 | 0 | 1 | 1 | 1 | 0 | 0 | 0 | 1 | 4 |
| Ganggayah et al (2021) | 0 | 0 | 0 | 0 | 1 | 0 | 1 | 1 | 0 | 0 | 3 |
| Pfob et al (2021) | 1 | 1 | 1 | 1 | 1 | 0 | 1 | 1 | 0 | 0 | 7 |
| Ueno et al (2022) | 1 | 0 | 0 | 1 | 1 | 0 | 1 | 1 | 0 | 0 | 5 |
| Peterson et al (2021) | 1 | 1 | 0 | 1 | 1 | 1 | 1 | 1 | 0 | 0 | 7 |
| Liu et al (2022) | 1 | 1 | 0 | 1 | 1 | 1 | 1 | 1 | 0 | 0 | 7 |
| Wang et al (2021) | 1 | 0 | 0 | 0 | 0 | 1 | 1 | 1 | 0 | 0 | 4 |
| Pinto et al (2022) | 1 | 1 | 0 | 1 | 1 | 0 | 1 | 1 | 0 | 0 | 6 |
| Agochukwu-Mmonu et al (2022) | 1 | 1 | 0 | 1 | 1 | 0 | 1 | 1 | 0 | 0 | 6 |
| Baglione et al (2022) | 1 | 0 | 1 | 1 | 1 | 0 | 1 | 1 | 0 | 0 | 6 |
| Noel et al (2022) | 1 | 1 | 1 | 1 | 1 | 1 | 1 | 1 | 1 | 0 | 9 |
| Van Dyk et al (2022) | 1 | 1 | 0 | 1 | 1 | 0 | 1 | 1 | 0 | 0 | 6 |
| Salima et al (2022) | 1 | 1 | 0 | 0 | 0 | 0 | 1 | 0 | 0 | 0 | 3 |
| Zeng et al (2022) | 1 | 1 | 0 | 1 | 1 | 0 | 1 | 0 | 1 | 0 | 6 |
| Hagiwara et al (2022) | 1 | 1 | 0 | 1 | 1 | 1 | 1 | 1 | 1 | 1 | 9 |
| Cunha et al (2022) | 1 | 1 | 1 | 1 | 1 | 0 | 1 | 1 | 0 | 0 | 7 |
| Xu et al (2022) | 1 | 1 | 1 | 1 | 1 | 1 | 1 | 1 | 1 | 1 | 10 |
| Parikh et al (2022) | 1 | 1 | 0 | 1 | 1 | 0 | 1 | 1 | 0 | 0 | 6 |
| Sidey-Gibbons et al (2022) | 1 | 1 | 1 | 1 | 1 | 0 | 1 | 1 | 1 | 0 | 8 |

Items for quality score:

I1: Research task is clearly stated, including the intended clinical problem, specified input predictors and an identified outcome

I2: The characteristics of the dataset (training and test sets if applicable) are detailed and have been shown to be representative to the research settings.

I3: Transformations of the data before it is applied to the proposed model are described (e.g., missing data imputation, normalization, feature selection, etc

I4: The validation methodology is clearly explained.

I5: The independence between training and test sets has been proven in the paper.

I6: Details on the models that were evaluated, and the adequate method developed to select the best model are provided

I7: An appropriate primary metric selected to evaluate algorithm performance (e.g.: AUC, F-score, etc.) has been clearly stated and reported.

I8: An adequate examination technique has been reported (e.g., feature importance, sensitivity analysis, saliency maps, etc.)

I9: A discussion of the reliability and robustness of the model is included and limitations for its clinical use have been clearly described.

I10: A link to the model development code, the final developed model or the data have been reported allowing replication.

Supplementary Table S 4 Study-level PRO & ML results combined

| *Author (year)* | *Country population* | *Cancer type* | *PRO outcome/feature* | *Data amount* | *Algorithm* | *CV train/ validation/test* | *Best result* | *Quality score (max 10)* | *Aim of the ML algorithm* |
| --- | --- | --- | --- | --- | --- | --- | --- | --- | --- |
| Walczak & Velanovich (2018) | US | pancreatic | feature | 219 | ANN | 2-fold CV | AUC = 0.657  ACC = 71.23% | 7 | Predict 7-month survival |
| Etminani-Ghasrodashti et al (2021) | US | unclear | outcome | 589 | LR; DT; RF; **ANN** | 80% train; 20% test | ACC = 69%  F1: 72% | 6 | Use environmental factors to predict QoL |
| Sharifi et al (2022) | Canada | prostate | outcome | 1214 | K-means+ ANN | 70% train; 15% validation; 15% test | R2 = 0.74 | 7 | Predict 1-year QoL |
| Nuutinen et al (2021) | Finland, Israel, Portugal, Italy | breast | outcome | 608 | RF | 90% train; 10% test | AUC = 0.832 | 6 | Predict 6-mont QoL |
| Etminani-Ghasrodashti et al (2021) | US | unclear | feature | 682 | LR; SVM; RF; **ANN** | 80% train; 20% test | ACC = 95%  F1 = 80% | 6 | Predict continuing/stopping treatment |
| Hatzilygeroudis et al (2021) | Greece | lung, prostate, breast, kidney (renal), intestine, cyst | feature | 43 | A total of 27 models and **their combination** | Leave-one-out CV | ACC = 93.02% | 5 | Predict 1-year prognosis of survival in patients with mtastatses trated with external beam radiation |
| Shi et al (2012) | Taiwan | breast | outcome | 402 | Linear regression; **ANN** | 80% train; 20% test | Body image: MAPE = 19.57%; Sexual functioning: MAPE = 8.79%; sexual enjoyment: MAPE = 8.84%; Future perspective: MAPE = 16.10% | 8 | Predict QoL (different outcomes) at 2 years after breast cancer surgery |
| Tsai et al (2013) | Taiwan | breast | outcome | 203 | Multiple regression; **ANN** | Not specified | **6 months**  PCS:  MAPE = 23.19%;  MCS: MAPE = 17.28%  **1 year**  PCS: MAPE = 15.32%;  MCS: MAPE = 16.22%  **2 years**  PCS: MAPE = 19.86%;  MCS: MAPE = 17.39% | 4 | Predict QoL (different outcomes) at 6 months, 1 year and 2 years after breast cancer surgery |
| Takehira et al (2011) | Japan | unclear | outcome | 40 | ANN | Leave-one-out CV | ACC = 60% | 4 | Predict QoL of cancer patients from evaluations of nurses and pharmacists |
| Cvetković (2017) | Serbia | breast | outcome | 84 | ANN | Not specified | RMSE = 0.853; R2 = 0.5343 | 2 | Prediction of depression |
| Lee et al (2018) | US | prostate | outcome | 324 | RF | 67% train; 33% test | AUC = 0.70 (for weak stream endpoint) | 5 | Predict a congenital genitourinary toxicity risk by using genome-wide single nucleotide polymorphism predictors |
| Chiu et al (2018) | Taiwan | hepatocellular | outcome | 332 | SVM; ANN; Multiple linear regression; Gaussian process regression | 132 train; 132 test; 68 external validation | Performance values reported for test/validation  FACT-Hep: MAPE = 4.4%/4.6%;  PCS: MAPE = 8.6%/5.4%;  MCS: MAPE =  5.2%/8.6% | 6 | Predict six-month QoL |
| Sasani et al (2019) | US | prostate, colorectal, gynecological, urologic, head & neck, hepatobiliary, thoracic | feature | 1901 | DT | Not specified | ACC = 78% | 4 | Based on components of geriatric assessment predict Timed Up and Go test results. |
| Lötsch et al (2018) | Finland | breast | both | 853 | RF | 67% train; 33% test | Balanced ACC = 63.7% | 4 | Create a simple questionnaire with a good predictive power for persisting pain after surgery |
| Sim & Yun (2019) | Korea | lung | feature | 809 | LR; DT; RF, Bagging; **AdaBoost** | 80% train; 20% test / 5-fold CV | ACC = 0.948 | 1 | Predict survival of lung cancer |
| Xu et al (2021) | China | colorectal, gastric, esophageal | both | 598 | LR; DT; **ANN** | 463 train; 135 test | ACC = 84.4% | 5 | Predict for postoperative fatigue after gastrointestinal tumor surgery. |
| Pan et al (2020) | US | prostate | outcome | 112 | RF; AdaBoost; **Gradient boosting decision tree** | 5-fold CV + test on external 26 | Urinary irritation: AUC = 0.79;  Urinary incontinence: AUC = 0.87;  On external cohort  Urinary irritation: AUC = 0.72;  Urinary incontinence: AUC = 0.77; | 6 | Predict 1-year changes in PROs from dosimetric parameters. |
| Juwara et al (2020) | Canada | breast | both | 204 | Least square regression; Ridge regression; Elastic net regression; RF; **Gradient boosting**; ANN | 80% train (with 10-fold CV) and 20% test | RMSE = 1.16 | 4 | Predict post-surgery neuropathic pain |
| Sim et al (2020) | Korea | lung | feature | 809 | LR; DT; **RF**; Bagging; AdaBoost | Both 5-fold CV and 80% train, 20% validation | AUC = 0.981 | 4 | Predict 5-year lung cancer survival from QoL data and sociodemographic data |
| Arkin et al (2020) | Türkiye | skin, lung, prostate, breast, colorectal, kidney (renal), bladder, gynecologcial, pancreatic, brain, laryngeal, pleural mesothelioma, gastric, esophageal | feature | 189 | LR; **ANN** | Done, but proportions not specified | AUC = 0.86 | 3 | Predict 30-days survival of cancer patients |
| Shafiei et al (2020) | US | colorectal, kidney (renal), bladder, gastric, esophageal | outcome | 25 | CNN+LSTM | Leave-one-supertrial-out cross-validation repeated in 5 folds. | HHI: ACC = 93.81%;  STAI: ACC = 94.76%;  WEMWBS: ACC = 95.0% | 4 | Predict mental health from eye movements |
| Yang et al (2021) | US | prostate | outcome | 52 | CNN autoencoder | 39 train (inside 5-fold CV); 13 test;  10 repetitions | ACC = 74% | 5 | Predict QoL from radiation treatment dose. |
| Lou et al (2020) | Taiwan | breast | feature | 1140 | k-NN; Naive-Bayes; SVM; **ANN**; Cox | Train = 798; Validation = 171; test = 171 | AUC = 0.976 | 5 | Predict 10-year recurrence after breast cancer surgery |
| Xuyi et al (2021) | Canada | lung, prostate, breast, colorectal, gynecological, head & neck, hematology, gastrointestinal, genitourinary | both | 46104 | ANN | 75% train; 15% test | Severe pain: AUC = 0.71; Moderate-severe depression: AUC = 0.73; Poor well-being: AUC = 0.70 | 5 | Predict risk of 6-month sever pain, moderate-severe depression and poor well-being after cancer diagnosis |
| Lee et al (2020) | US, France | breast | both | 2799 | Preconditioned RF regression | Overall fatigue: 377 train, 161 test;  Physical fatigue: 283 train, 121 test; Emotional fatigue: 515 train, 220 test; Cognitive fatigue: 820 train, 21 test | Cognitive fatigue: AUC = 0.60  All others AUC<0.50 | 6 | Predict 1-year fatigue after breast cancer treatment |
| Kourou et al (2021) | Finland, Israel, Portugal, Italy | breast | both | 609 | **SVM**, RF, Gradient boosting | 80% train (5-fold CV), 20% test | Balanced ACC = 82.5% | 8 | Predict mental health outcomed following breast cancer diagnosis |
| Rossi et al (2021) | US | lung, gastrointestinal | feature | 52 | LR | Nested cross validation: 4-fold patient cross validation (inner loop), leave-one-out cross validation (outer loop) | AUC = 0.74 | 7 | Predict post-discharge compications |
| Wang et al (2021) | China | lung | both | 746 | SVM, **DT**, Bayesian neural network | 513 train (inner 10-fold CV), 108 test, 125 external validation | AUC = 0.88 in external validation | 5 | Predict whether patients would receive or not local treatment. |
| Sidey-Gibbons et al (2021) | US | breast | outcome | 611 | SVM, DT, Elastic net, ANN, **ensemble** | 67% train, 33% trst | AUC = 0.85 | 8 | Predict an individual’s risk of financial toxicity |
| Haun et al (2021) | Germany | prostate, breast, colorectal | both | 496 | RF,  least square, Ridge regression, **LASSO**, Elastic net, XGBoost | 70% train (inner 10-fold CV), 30% test | RMSE = 0.370 | 8 | Prediction of anxiety in cancer survivors |
| Wakabayashi et al (2021) | Japan | lung, breast, digestive, head & neck | both | 69 | RF | Leave-one-out CV | AUC = 0.848 | 6 | Predict pain response/relief after radiotherapy for spinal metastases. |
| Iivanainen et al (2021) | Finland | skin, lung, genitourinary, head & neck | feature | 34 | XGboost | 70% train (inner 5-fold CV), 30% test | Presence: AUC = 0.99; Onset: AUC = 0.67 | 4 | Predict presence and onset of immune-related adverse events |
| Kober et al (2021) | US | lung, breast, gynecological, gastrointestinal | both | 1217 | SVM, **RF**, CART | 10-fold CV | RMSE = 1.53 | 8 | Predict severity of evening fatigue the week following chemotherapy |
| Crumpei-Tanasă & Crumpei (2021) | Romania | breast | feature | 70 | k-NN, LR, Naive Bayes, SVM, DT, LDA, **Gradient boosting** | 10-fold CV | Stress hormones: ACC = 81.2%; Inflammatory markers: ACC = 70% | 4 | Predict stress hormones and inflammatory markers in breast cancer survivor |
| Ganggayah et al (2021) | Malaysia | breast | feature | 1000 | SVM, DT, **RF** | 70% train, 30% test | ACC = 92.5% | 3 | Predict survival |
| Pfob et al (2021) | US, Canada | breast | both | 1553 | LR, **ANN**, XGBoost | 1332 train (inner 10-fold CV), 221 test | Lower satisfaction: AUC = 0.85;  Higher satisfaction: AUC = 0.87 | 7 | Predict patient-reported satisfaction with breast at 2-year follow-up in women undergoing cancer-related mastectomy |
| Ueno et al (2022) | Japan | breast | both | 759 | **LR**, XGboost | 8-fold CV | AUC = 0.76 | 5 | Predict insomnia |
| Peterson et al (2021) | US | breast, gynecological, gastrointestinal, lymphoma, genitourinary, thoracic, head & neck | feature | 8439 | k-NN, LR, SVM, RF, Gradient boosted tree, ANN, **ensemble** | 80% train, 20% test | AUC = 0.806 | 7 | Predict risk of preventable acute care use after chemotherapy |
| Liu et al (2022) | China | thyroid | outcome | 286 | RF | 70% train, 30% test | AUC = 0.897 | 7 | Predict QoL after thyroidectomy |
| Wang et al (2021) | US | head & neck | both | 823 | LSTM | Not specified | At 6 weeks: RMSE = 1.91; At 12 months: RMSE = 1.42 | 4 | Prediction of symptoms after treatment |
| Pinto et al (2022) | Italy | skin | outcome | 203 | RF | 62 train, 36 validation, 44 test | MSE = 0.45; R2 = 0.78 | 6 | Predict QoL from demographic and clinical characteristics |
| Agochukwu-Mmonu et al (2022) | US | prostate | both | 3983 | Gradient-boosting DT | 2653 train, 1330 test, 837 validation | Sexual domain at 12 months:  AUC = 0.91;  Sexual domain at 24 months:  AUC = 0.94;  Erection quality at 12 months: AUC = 0.89; Erection quality at 24 months: AUC = 0.92 | 6 | Predict sexual recovery after radical prostatectomy |
| Baglione et al (2022) | US | breast | outcome | 40 | **RF**, XGBoost | Leave-one-out CV | ACC =  84.6% | 6 | Mood prediction via app engagement. |
| Noel et al (2022) | Canada | head & neck | feature | 11761 | K-NN. LR, RF, **Gradient boosting machine**, ANN | 80% train, 20% test. 5-fold CV for hyperparameter selection | AUC = 0.80 | 9 | Predict Emergency Department Use and Unplanned Hospitalization in Patients |
| Van Dyk et al (2022) | US | breast | both | 876 | LR (**LASSO** and Elastic Net), RF, Stochastic gradient boosting | Leave-one-out CV | AUC = 0.736 (vs controls)  AUC = 0.744 (vs survivors without decline) | 6 | Identify patients with persistent self-reported cognitive decline |
| Salima et al (2022) | Indonesia | gynecological | feature | 115 | DT | Not specified | RR ratio = 0.682 | 3 | Early detection of ovarian cancer |
| Zeng et al (2022) | China | lung | feature | 1106 | LASSO + Cox regression | 75% train, 25% test | C-index = 0.789 | 6 | Survival prediction in home hospice care patients |
| Hagiwara et al (2022) | Japan | lung, breast, colorectal, stomach | both | 903 | Gradient-boosted tree | 80% train, 20% test | RMSE = 0.025 | 9 | Mapping Cancer Quality of Life Questionnaire Core 30 Onto 5-Level Version of EQ-5D Index for Patients With Cancer |
| Cunha et al (2022) | Brazil | lung | feature | 148 | Extra Trees classifier | 80% train, 20% validation, test external 42 patients | C-statistic = 0.706; ACC = 71.4% | 7 | Predict 90-day survival |
| Xu et al (2022) | US | unclear | feature | 630 | K-NN, SVM, DT, **XGBoost**, generalized regression, ANN, multivariate spline | 80% train, 20% test | AUC = 0.69 | 10 | Predict 180-day mortality |
| Parikh et al (2022) | US | skin, breast, thyroid, gynecological, leukemia, gastrointestinal, thoracic, genitourinary, lymphoma, myeloma | feature | 5870 | LR | 70% train, 30% test | AUC = 0.86 | 6 | Predict 6-month mortality |
| Sidey-Gibbons et al (2022) | US | gynecological | feature | 243 | LR, SVM, Generalized additive model, Boosted trees, ANN, Multivariate adaptive regression spline and **ensemble of all** | Train and test in 2:1 ratio | ACC = 0.79 | 8 | Predict 180-day mortality |

**Performance measures**: *AUC*: area under the receiver operating characteristic curve, *ACC*: accuracy, *F1*: F1-score, *R2*: Coefficient of determination, *MAPE*: mean absolute percentage error, *RMSE:* root mean square error, *RR-ratio*: risk/reward ratio, *C-index/C-statistic*: concordance index/statistic

**Outcomes**: *PCS*:physical component summary, *MCS*:mental component summary, *FACT-Hep*: Functional Assessment of Cancer Therapy – Hepatobiliary, HHI: Herth hope index, STAI: state trait anxiety inventory, WEMWBS: Waraick Edinburgh Mental Wellbeing Scale,

**Algorithms**: *ANN*:artificial neural network, *LR*: logistic regression, *DT*: decision tree, *RF*:random forest, *SVM*: support vector machine, *AdaBoost*: adaptive boosting, *CNN*:convolutional neural network, *LSTM:* long-short term memory, *CART* = classification and regression tree, *k-NN*: k nearest neighbour, *LDA*: linear discriminant analysis, *LASSO*: least absolute shrinkage and selection operator, *XGBoost*: extreme gradient boosting,

**Other abbreviations***: PRO*: patient reported outcome, *CV*: cross-validation, *QoL*:quality of life

**References of studies included in the review (following the order of the table)**

S. Walczak and V. Velanovich, ‘Improving prognosis and reducing decision regret for pancreatic cancer treatment using artificial neural networks’, Decision Support Systems, vol. 106, pp. 110–118, Feb. 2018, doi: 10.1016/j.dss.2017.12.007.

R. Etminani-Ghasrodashti, C. Kan, M. Arif Qaisrani, O. Mogultay, and H. Zhou, ‘Examining the Impacts of the Built Environment on Quality of Life in Cancer Patients Using Machine Learning’, Sustainability, vol. 13, no. 10, p. 5438, May 2021, doi: 10.3390/su13105438.

F. Sharifi, E. Mohammed, T. Crump, and B. H. Far, ‘Explainable Analytics to Predict the Quality of Life in Patients with Prostate Cancer from Longitudinal Data’, Applied Artificial Intelligence, vol. 36, no. 1, p. 2055393, Dec. 2022, doi: 10.1080/08839514.2022.2055393.

M. Nuutinen et al., ‘Impact of Machine Learning Assistance on the Quality of Life Prediction for Breast Cancer Patients’:, in Proceedings of the 15th International Joint Conference on Biomedical Engineering Systems and Technologies, Online Streaming, --- Select a Country ---: SCITEPRESS - Science and Technology Publications, 2022, pp. 344–352. doi: 10.5220/0010786900003123.

R. Etminani-Ghasrodashti, C. Kan, and L. Mozaffarian, ‘Investigating the Role of Transportation Barriers in Cancer Patients’ Decision Making Regarding the Treatment Process’, Transportation Research Record, vol. 2675, no. 6, pp. 175–187, Jun. 2021, doi: 10.1177/0361198121991497.

I. Hatzilygeroudis and J. Prentzas, ‘AI Approaches for the Prognosis of the Survival (or Not) of Patients with Bone Metastases’, in 2021 IEEE 33rd International Conference on Tools with Artificial Intelligence (ICTAI), Washington, DC, USA: IEEE, Nov. 2021, pp. 1353–1357. doi: 10.1109/ICTAI52525.2021.00215.

HY Shi, JT Tsai, YM Chen, R Culbertson, HT Chang, and MF Hou, ‘Predicting two-year quality of life after breast cancer surgery using artificial neural network and linear regression models.’, Breast cancer research and treatment, vol. 135, no. 1, Aug. 2012, doi: 10.1007/s10549-012-2174-6.

JT Tsai, MF Hou, YM Chen, TT Wan, HY Kao, and HY Shi, ‘Predicting quality of life after breast cancer surgery using ANN-based models: performance comparison with MR.’, Supportive care in cancer : official journal of the Multinational Association of Supportive Care in Cancer, vol. 21, no. 5, May 2013, doi: 10.1007/s00520-012-1672-8.

R Takehira, K Murakami, S Katayama, K Nishizawa, and S Yamamura, ‘Artificial Neural Network Modeling of Quality of Life of Cancer Patients: Relationships between Quality of Life Assessments, as Evaluated by Patients, Pharmacists, and Nurses.’, International journal of biomedical science : IJBS, vol. 7, no. 4, Dec. 2011.

J Cvetković, ‘Breast Cancer Patients’ Depression Prediction by Machine Learning Approach.’, Cancer investigation, vol. 35, no. 8, Sep. 2017, doi: 10.1080/07357907.2017.1363892.

J Lötsch, A Ultsch, and E Kalso, ‘Prediction of persistent post-surgery pain by preoperative cold pain sensitivity: biomarker development with machine-learning-derived analysis.’, British journal of anaesthesia, vol. 119, no. 4, Oct. 2017, doi: 10.1093/bja/aex236.

S Lee, S Kerns, H Ostrer, B Rosenstein, JO Deasy, and JH Oh, ‘Machine Learning on a Genome-wide Association Study to Predict Late Genitourinary Toxicity After Prostate Radiation Therapy.’, International journal of radiation oncology, biology, physics, vol. 101, no. 1, May 2018, doi: 10.1016/j.ijrobp.2018.01.054.

CC Chiu et al., ‘Comparison of Models for Predicting Quality of Life After Surgical Resection of Hepatocellular Carcinoma: a Prospective Study.’, Journal of gastrointestinal surgery : official journal of the Society for Surgery of the Alimentary Tract, vol. 22, no. 10, Oct. 2018, doi: 10.1007/s11605-018-3833-7.

K Sasani et al., ‘Gait speed and survival of older surgical patient with cancer: Prediction after machine learning.’, Journal of geriatric oncology, vol. 10, no. 1, Jan. 2019, doi: 10.1016/j.jgo.2018.06.012.

JA Sim and YH Yun, ‘Predicting Disease-Free Lung Cancer Survival Using Patient Reported Outcome (PRO) Measurements with Comparisons of Five Machine Learning Techniques (MLT).’, Studies in health technology and informatics, vol. 264, Aug. 2019, doi: 10.3233/SHTI190548.

XY Xu, JL Lu, Q Xu, HX Hua, L Xu, and L Chen, ‘Risk factors and the utility of three different kinds of prediction models for postoperative fatigue after gastrointestinal tumor surgery.’, Supportive care in cancer : official journal of the Multinational Association of Supportive Care in Cancer, vol. 29, no. 1, Jan. 2021, doi: 10.1007/s00520-020-05483-0.

X Pan et al., ‘Dosimetric predictors of patient-reported toxicity after prostate stereotactic body radiotherapy: Analysis of full range of the dose-volume histogram using ensemble machine learning.’, Radiotherapy and oncology : journal of the European Society for Therapeutic Radiology and Oncology, vol. 148, Jul. 2020, doi: 10.1016/j.radonc.2020.04.013.

L Juwara, N Arora, M Gornitsky, P Saha-Chaudhuri, and AM Velly, ‘Identifying predictive factors for neuropathic pain after breast cancer surgery using machine learning.’, International journal of medical informatics, vol. 141, Sep. 2020, doi: 10.1016/j.ijmedinf.2020.104170.

JA Sim et al., ‘The major effects of health-related quality of life on 5-year survival prediction among lung cancer survivors: applications of machine learning.’, Scientific reports, vol. 10, no. 1, Jul. 2020, doi: 10.1038/s41598-020-67604-3.

FS Arkin, G Aras, and E Dogu, ‘Comparison of Artificial Neural Networks and Logistic Regression for 30-days Survival Prediction of Cancer Patients.’, Acta informatica medica : AIM : journal of the Society for Medical Informatics of Bosnia & Herzegovina : casopis Drustva za medicinsku informatiku BiH, vol. 28, no. 2, Jun. 2020, doi: 10.5455/aim.2020.28.108-113.

SB Shafiei, Z Lone, AS Elsayed, AA Hussein, and KA Guru, ‘Identifying mental health status using deep neural network trained by visual metrics.’, Translational psychiatry, vol. 10, no. 1, Dec. 2020, doi: 10.1038/s41398-020-01117-5.

Z Yang et al., ‘Machine learning and statistical prediction of patient quality-of-life after prostate radiation therapy.’, Computers in biology and medicine, vol. 129, Feb. 2021, doi: 10.1016/j.compbiomed.2020.104127.

SJ Lou et al., ‘Machine Learning Algorithms to Predict Recurrence within 10 Years after Breast Cancer Surgery: A Prospective Cohort Study.’, Cancers, vol. 12, no. 12, Dec. 2020, doi: 10.3390/cancers12123817.

W Xuyi, H Seow, and R Sutradhar, ‘Artificial neural networks for simultaneously predicting the risk of multiple co-occurring symptoms among patients with cancer.’, Cancer medicine, vol. 10, no. 3, Feb. 2021, doi: 10.1002/cam4.3685.

S Lee et al., ‘Prediction of Breast Cancer Treatment-Induced Fatigue by Machine Learning Using Genome-Wide Association Data.’, JNCI cancer spectrum, vol. 4, no. 5, Oct. 2020, doi: 10.1093/jncics/pkaa039.

K Kourou et al., ‘A machine learning-based pipeline for modeling medical, socio-demographic, lifestyle and self-reported psychological traits as predictors of mental health outcomes after breast cancer diagnosis: An initial effort to define resilience effects.’, Computers in biology and medicine, vol. 131, Apr. 2021, doi: 10.1016/j.compbiomed.2021.104266.

LA Rossi, LG Melstrom, Y Fong, and V Sun, ‘Predicting post-discharge cancer surgery complications via telemonitoring of patient-reported outcomes and patient-generated health data.’, Journal of surgical oncology, vol. 123, no. 5, Apr. 2021, doi: 10.1002/jso.26413.

Z Wang et al., ‘Machine Learning Algorithm Guiding Local Treatment Decisions to Reduce Pain for Lung Cancer Patients with Bone Metastases, a Prospective Cohort Study.’, Pain and therapy, vol. 10, no. 1, Jun. 2021, doi: 10.1007/s40122-021-00251-2.

C Sidey-Gibbons et al., ‘Development of Machine Learning Algorithms for the Prediction of Financial Toxicity in Localized Breast Cancer Following Surgical Treatment.’, JCO clinical cancer informatics, vol. 5, Mar. 2021, doi: 10.1200/CCI.20.00088.

MW Haun, L Simon, H Sklenarova, V Zimmermann-Schlegel, HC Friederich, and M Hartmann, ‘Predicting anxiety in cancer survivors presenting to primary care - A machine learning approach accounting for physical comorbidity.’, Cancer medicine, vol. 10, no. 14, Jul. 2021, doi: 10.1002/cam4.4048.

K Wakabayashi et al., ‘A predictive model for pain response following radiotherapy for treatment of spinal metastases.’, Scientific reports, vol. 11, no. 1, Jun. 2021, doi: 10.1038/s41598-021-92363-0.

S Iivanainen, J Ekstrom, H Virtanen, VV Kataja, and JP Koivunen, ‘Electronic patient-reported outcomes and machine learning in predicting immune-related adverse events of immune checkpoint inhibitor therapies.’, BMC medical informatics and decision making, vol. 21, no. 1, Jun. 2021, doi: 10.1186/s12911-021-01564-0.

KM Kober et al., ‘Prediction of evening fatigue severity in outpatients receiving chemotherapy: less may be more.’, Fatigue : biomedicine, health & behavior, vol. 9, no. 1, 2021, doi: 10.1080/21641846.2021.1885119.

I Crumpei-Tanasă and I Crumpei, ‘A Machine Learning Approach to Predict Stress Hormones and Inflammatory Markers Using Illness Perception and Quality of Life in Breast Cancer Patients.’, Current oncology (Toronto, Ont.), vol. 28, no. 4, Aug. 2021, doi: 10.3390/curroncol28040275.

MD Ganggayah et al., ‘An Artificial Intelligence-Enabled Pipeline for Medical Domain: Malaysian Breast Cancer Survivorship Cohort as a Case Study.’, Diagnostics (Basel, Switzerland), vol. 11, no. 8, Aug. 2021, doi: 10.3390/diagnostics11081492.

A Pfob, BJ Mehrara, JA Nelson, EG Wilkins, AL Pusic, and C Sidey-Gibbons, ‘Machine learning to predict individual patient-reported outcomes at 2-year follow-up for women undergoing cancer-related mastectomy and breast reconstruction (INSPiRED-001).’, Breast (Edinburgh, Scotland), vol. 60, Dec. 2021, doi: 10.1016/j.breast.2021.09.009.

T Ueno et al., ‘Comorbid insomnia among breast cancer survivors and its prediction using machine learning: a nationwide study in Japan.’, Japanese journal of clinical oncology, vol. 52, no. 1, Jan. 2022, doi: 10.1093/jjco/hyab169.

DJ Peterson, NP Ostberg, DW Blayney, JD Brooks, and T Hernandez-Boussard, ‘Machine Learning Applied to Electronic Health Records: Identification of Chemotherapy Patients at High Risk for Preventable Emergency Department Visits and Hospital Admissions.’, JCO clinical cancer informatics, vol. 5, Oct. 2021, doi: 10.1200/CCI.21.00116.

YH Liu, J Jin, and YJ Liu, ‘Machine learning-based random forest for predicting decreased quality of life in thyroid cancer patients after thyroidectomy.’, Supportive care in cancer : official journal of the Multinational Association of Supportive Care in Cancer, vol. 30, no. 3, Mar. 2022, doi: 10.1007/s00520-021-06657-0.

Y Wang et al., ‘Predicting late symptoms of head and neck cancer treatment using LSTM and patient reported outcomes.’, Proceedings. International Database Engineering and Applications Symposium, vol. 2021, Jul. 2021, doi: 10.1145/3472163.3472177.

M Pinto, N Marotta, C Caracò, E Simeone, A Ammendolia, and A de Sire, ‘Quality of Life Predictors in Patients With Melanoma: A Machine Learning Approach.’, Frontiers in oncology, vol. 12, 2022, doi: 10.3389/fonc.2022.843611.

N Agochukwu-Mmonu et al., ‘Development and Validation of Dynamic Multivariate Prediction Models of Sexual Function Recovery in Patients with Prostate Cancer Undergoing Radical Prostatectomy: Results from the MUSIC Statewide Collaborative.’, European urology open science, vol. 40, Jun. 2022, doi: 10.1016/j.euros.2022.03.009.

AN Baglione, L Cai, A Bahrini, I Posey, M Boukhechba, and PI Chow, ‘Understanding the Relationship Between Mood Symptoms and Mobile App Engagement Among Patients With Breast Cancer Using Machine Learning: Case Study.’, JMIR medical informatics, vol. 10, no. 6, Jun. 2022, doi: 10.2196/30712.

CW Noel et al., ‘Development and Validation of a Machine Learning Algorithm Predicting Emergency Department Use and Unplanned Hospitalization in Patients With Head and Neck Cancer.’, JAMA otolaryngology-- head & neck surgery, vol. 148, no. 8, Aug. 2022, doi: 10.1001/jamaoto.2022.1629.

K Van Dyk et al., ‘Associating persistent self-reported cognitive decline with neurocognitive decline in older breast cancer survivors using machine learning: The Thinking and Living with Cancer study.’, Journal of geriatric oncology, vol. 13, no. 8, Nov. 2022, doi: 10.1016/j.jgo.2022.08.005.

S Salima et al., ‘Ovarian Cancer-Self Assessment: An Innovation for Early Detection and Risk Assessment of Ovarian Cancer.’, Asian Pacific journal of cancer prevention : APJCP, vol. 23, no. 8, Aug. 2022, doi: 10.31557/APJCP.2022.23.8.2643.

Y Zeng et al., ‘Survival Prediction in Home Hospice Care Patients with Lung Cancer Based on LASSO Algorithm.’, Cancer control : journal of the Moffitt Cancer Center, vol. 29, 2022, doi: 10.1177/10732748221124519.

Y Hagiwara et al., ‘Gradient Boosted Tree Approaches for Mapping European Organization for Research and Treatment of Cancer Quality of Life Questionnaire Core 30 Onto 5-Level Version of EQ-5D Index for Patients With Cancer.’, Value in health : the journal of the International Society for Pharmacoeconomics and Outcomes Research, Sep. 2022, doi: 10.1016/j.jval.2022.07.020.

MT Cunha, AP de Souza Borges, V Carvalho Jardim, A Fujita, and G de Castro Jr, ‘Predicting survival in metastatic non-small cell lung cancer patients with poor ECOG-PS: A single-arm prospective study.’, Cancer medicine, Sep. 2022, doi: 10.1002/cam4.5254.

C Xu, IM Subbiah, SC Lu, A Pfob, and C Sidey-Gibbons, ‘Machine learning models for 180-day mortality prediction of patients with advanced cancer using patient-reported symptom data.’, Quality of life research : an international journal of quality of life aspects of treatment, care and rehabilitation, Oct. 2022, doi: 10.1007/s11136-022-03284-y.

RB Parikh et al., ‘Development of Machine Learning Algorithms Incorporating Electronic Health Record Data, Patient-Reported Outcomes, or Both to Predict Mortality for Outpatients With Cancer.’, JCO clinical cancer informatics, vol. 6, Dec. 2022, doi: 10.1200/CCI.22.00073.

CJ Sidey-Gibbons et al., ‘Predicting 180-day mortality for women with ovarian cancer using machine learning and patient-reported outcome data.’, Scientific reports, vol. 12, no. 1, Dec. 2022, doi: 10.1038/s41598-022-22614-1.
